# Association of HLA-DPB1 with Scleroderma and Its Clinical Features in Chinese Population

**DOI:** 10.1371/journal.pone.0087363

**Published:** 2014-01-31

**Authors:** Jiucun Wang, Xinjian Guo, Lin Yi, Gang Guo, Wenzhen Tu, Wenyu Wu, Li Yang, Rong Xiao, Yuan Li, Haiyan Chu, Dongyi He, Li Jin, Maureen D. Mayes, Hejian Zou, Xiaodong Zhou

**Affiliations:** 1 Ministry of Education (MOE) Key Laboratory of Contemporary Anthropology and State Key Laboratory of Genetic Engineering, School of Life Sciences, Fudan University, Shanghai, China; 2 Institute of Rheumatology, Immunology and Allergy, Fudan University, Shanghai, China; 3 Division of Rheumatology and Clinical Immunogenetics, The University of Texas Medical School at Houston, Houston, Texas, United States of America; 4 Gansu College of Traditional Chinese Medicine, Lanzhou, Gansu, China; 5 Yiling Hospital, Shijiazhuang, Hebei Province, China; 6 Shanghai Traditional Chinese Medicine-Integrated Hospital, Shanghai, China; 7 Division of Dermatology, Huashan Hospital, Fudan University, Shanghai, China; 8 Division of Rheumatology, Teaching Hospital of Chengdu University of TCM, Chengdu, Sichuan Province, China; 9 Department of Dermatology, Second Xiangya Hospital, Central South University, Changsha, Hunan Province, China; 10 Department of Rheumatology, Shanghai Guanghua Hospital, Shanghai, China; 11 Division of Rheumatology, Huashan Hospital, Fudan University, Shanghai, China; Keio University School of Medicine, Japan

## Abstract

Human leukocyte antigen DPB1 was reported to contain singly nucleotide polymorphisms conferring the strongest susceptibility to systemic sclerosis in Korean population. However, associations of specific DPB1 alleles with SSc vary in different ethnic populations. The aim of this study was to profile DPB1 alleles in Chinese population and to identify specific DPB1 alleles in association with SSc and clinical and serological features of SSc in Han Chinese. A cohort containing 338 patients with SSc and 480 gender-matched and unrelated controls were examined in the study. The HLA-DPB1 genotyping was performed with sequence-based typing method. Exact p-values were obtained (Fisher's test) from 2×2 tables of allele counts or allele carriers and disease status. Thirty eight DPB1 alleles were found in the cohort. DPB1*05:01 was the most common allele in this cohort. DPB1*03:01 and *13:01 were significantly increased in SSc. DPB1*13:01 association had already been described in other ethnic populations, whereas DPB1*03:01 was specific to Han Chinese patients with SSc. In addition, comparisons between SSc subsets indicated that patients carrying DPB1*03:01 were more likely to develop pulmonary fibrosis, DPB1*04 carriers were increased in SSc patients with anti-centromere autoantibodies and in contrast, SSc patients with homozygous DPB1*05:01 showed an opposite association with marginal significance.

## Introduction

Systemic sclerosis (SSc) or scleroderma is a rare and complex immune-mediated disease. It is clinically characterized by fibrosis of skin and internal organs. Based on the extent of skin fibrosis, SSc can be classified into two clinical subsets: limited cutaneous (lcSSc) and diffuse cutaneous (dcSSc) SSc. The latter subset is characterized by more rapid progression of skin and visceral involvement, as well as poorer prognosis [1]. SSc patients also are characterized by the presence of a group of autoantibodies in circulation, and which classify SSc patients into specific autoantibody subsets. The most common of these autoantibodies are directed against DNA topoisomerase I (ATA), centromeric proteins (ACA) and RNA polymerases III (anti-RNAP3) [2], [3].

Genetic studies of SSc indicated that specific genotypes of multiple genes contributing to susceptibility to SSc and its clinical presentations [4], [5], [6]. A recent genome wide association study (GWAS) in a Korean SSc cohort revealed that the specific single nucleotide polymorphisms (SNPs) of human leukocyte antigen (HLA) DPB1 conferred the strongest susceptibility to SSc, and HLA-DPB1*13:01 and DPB1*09:01 were the most susceptible alleles to Korean SSc [6]. Studies of US Caucasian population supported DPB1*13:01, but not DPB1*09:01, as a major susceptibility allele to SSc, and especially to ATA positive SSc [7], which was consistent with the reports of a SSc cohort of UK Caucasians [8]. Studies of South Africans indicated that DPB1*13:01 was significantly increased in dcSSc, but not in lcSSc [9]. On the other hand, a Japanese SSc cohort supported only DPB1*09:01 in association with SSc [10]. However, African-American and Hispanic SSc of US cohorts did not show any association with HLA-DPB1 [7].

Chinese SSc patients have unique serological and clinical features with high frequency of ATA, dcSSc and pulmonary fibrosis but low in anti-RNAP3 [11]. Associations between the HLA alleles and SSc have not been reported in Chinese SSc. Recently, we established a SSc cohort of Han Chinese through multicenter SSc consortium in China under the International Network of Scleroderma Clinical Care and Research (InSCAR) (http://www.inscar-global.org). The goal of the present study is to investigate the HLA-DPB1 alleles in association with potential risk to or protection from SSc in Han Chinese.

## Materials and Methods

### Patient enrollment

SSc patients were recruited from a multicenter study including hospitals and outpatient clinics in Shanghai, Hebei province, Sichuan province, and Hunan province in China. All patients met the American College of Rheumatology (ACR) classification criteria for SSc [12], or had at least 3 out of 5 CREST features (Calcinosis, Raynaud's phenomenon, Esophageal dysmotility, Sclerodactyly, and Telangiectasia) with sclerodactyly being mandatory [13]. A total of 338 patients with SSc and 480 gender-matched and unrelated controls were examined in the studies. None of the controls had autoimmune diseases. There were 129 lcSSc and 159 dcSSc, others were undefined. The studies were approved by the institutional review boards of the University of Texas Health Science Center at Houston, United States of American and Fudan University, Shanghai, China. Written informed consent was obtained from each study subject.

### Autoantibodies Tests

Patient's sera were tested for antinuclear antibodies (ANA) by indirect immunofluorescence using HEp-2 cells as antigen substrate (Antibodies, Davis, CA). ATA was detected by passive immunodiffusion against calf thymus extracts (INOVA, Diagnostics). ACA was determined by indirect immunofluorescence using HEp-2 cells. Anti-RNAP3 was detected utilizing commercially available kits (NBL, Nagoya, Japan).

### HLA-DPB1 genotyping

Genomic DNA was extracted from peripheral blood cells from subjects. The HLA-DPB1 genotyping was performed with sequence-based typing (SBT) method using SeCore Kits (Life Technologies, USA). Briefly, the allele-specific polymerase chain reactions (PCR) were performed using primers supplied in the SeCore kits, and then were followed by sequencing exon 2, 3 and 4 of the HLA-DPB1 gene, as well as two additional targeted sequencing on codon 2 and 85. The HLA SBT uTYPE 6.0 program (Life Technologies) was used in sequencing analysis and assigning HLA-DPB1 alleles.

### Statistical Analysis

Exact p-values were obtained (Fisher's test) from 2×2 tables of allele counts and disease status. The p values less than 0.05 were considered nominal significance. Here we apply both nominal significance and a strict “Bonferroni” correction to the p-values, and allow the reader to consider the context. Due to extensive and long-range haplotypes in the HLA-DPB1 region, a Bonferroni correction is highly conservative.

## Results

Autoantibody tests showed that 93.3% SSc patients were ANA positive. There were 304 patients examined for ATA with 142 being positive (46.7%), 272 were examined for ACA with 38 positive (14%), 251 were examined for anti-RNAP3 with 7 positive (2.8%). Out of 277 patients who were examined with chest X-ray and/or CT, 199 were diagnosed as pulmonary fibrosis (71.8%).

A total of thirty eight DPB1 alleles were found in the cases and/or controls. Fourteen of them were observed with frequency over 1%, and that was listed in the Table 1. Bonferroni correction for significance was calculated as P<0.0013.

**Table 1:** Distribution of major HLA-DPB1 alleles in Chinese controls and SSc patients.

	Control	%	SSc	%	*p*	OR (95% CI)
DPB1*02:01	**187**	**19.48**	**95**	**14.05**	**0.0042**	**0.68 (0.51–0.89)**
DPB1*02:02	64	6.67	42	6.21	0.71	0.93 (0.61–1.41)
DPB1*03:01	**27**	**2.81**	**52**	**7.69**	**5.8×10^−6^**	**2.88 (1.75–4.76)**
DPB1*04:01	85	8.85	48	7.10	0.2	0.79 (0.54–1.15)
DPB1*04:02	37	3.85	19	2.81	0.25	0.72 (0.4–1.31)
DPB1*05:01	362	37.71	263	38.91	0.62	1.05 (0.85–1.29)
DPB1*09:01	15	1.56	8	1.18	0.52	0.75 (0.29–1.9)
DPB1*13:01	**43**	**4.48**	**59**	**8.73**	**0.00047**	**2.04 (1.33–3.12))**
DPB1*14:01	18	1.88	11	1.63	0.71	0.87 (0.38–1.94)
DPB1*17:01	**50**	**5.21**	**18**	**2.66**	**0.011**	**0.5 (0.28–0.89)**
DPB1*19:01	9	0.94	7	1.04	0.84	1.11 (0.37–3.25)
DPB1*21:01	19	1.98	9	1.33	0.32	0.67 (0.28–1.57)
DPB1*35:01	**2**	**0.21**	**7**	**1.04**	**0.026**	**5.01 (0.96–34.97)**
DPB1*135:01	8	0.83	10	1.48	0.22	1.79 (0.65–4.98)
Others		3.54		4.14		
Total	960	100	676	100		

OR = odds ratio; CI =  confidence interval; nominal significance *p* value<0.05; Bonferroni correction for significance was calculated as *p*<0.0013.

HLA-DPB1*05:01 appeared the most common in the Chinese cohort (37.7% in controls and 38.9% in cases), and was followed by DPB1*02:01 that was decreased in SSc patients (19.5% in controls vs. 14.1% in cases, p = 0.0042). DPB1*17:01 also was decreased in Chinese SSc (5.2% in controls vs. 2.7% in cases, p = 0.011). On the other hand, DPB1*13:01 and DPB1*03:01 were significantly increased in Chinese SSc that passed Bonferroni correction (frequency of alleles in controls vs. cases and p value were 4.48 vs. 8.73%, 0.00047, 2.81% vs. 7.69%, 5.8×10^−6^, respectively) (Table 1). In addition, DPB1*35:01 also was increased in SSc, and passed nominal significance level (1.04% in SSc vs. 0.21% in controls, p = 0.026). Male and female groups did not show significant difference in allele association with SSc.

Comparisons between controls and SSc subsets showed that DPB1*03:01was significantly increased in frequency in lcSSc, dcSSc, ATA positive and ACA positive SSc, as well as in SSc with pulmonary fibrosis (PF); DPB1*13:01 showed similar increases, except not in ACA positive SSc. In addition, at nominal significance level, DPB1*35:01 was increased only in ACA positive and PF negative SSc. On the other hand, a decreased frequency of DPB1*02:01 showed in lcSSc, ATA positive SSc and SSc patients with PF, and a decreased DPB1*17:01 in dcSSc and SSc with PF, which also achieved a nominal significance (p<0.05) (Table 2). Of notes, the number of SSc patients with anti-RNAP3 autoantibodies was under power in the studies of SSc subsets.

**Table 2:** Comparisons between controls and SSc subsets.

	Alleles	DPB1*02:01	DPB1*03:01	DPB1*13:01	DPB1*17:01	DPB1*35:01
**lcSSc**	*p*	**0.02**	**2.6×10^−4^**	**0.019**	0.38	0.33
	OR (95% CI)	0.63 (0.41–0.95)	2.9 (1.54–5.47)	1.89 (1.06–3.34)	0.73 (0.34–1.52)	5.64 (0.77–48.3)
**dcSSc**	*p*	0.08	**7.8×10^−5^**	**9.7×10^−4^**	**0.024**	0.25
	OR (95% CI)	0.73 (0.51–1.05)	2.95 (1.63–5.34)	2.22 (1.33–3.7)	0.41 (0.17–0.95)	3.03 (0.31–30.1)
**ATA (+)**	*p*	**0.027**	**1×10^−7^**	**1.3×10^−4^**	0.052	**0.05**
	OR (95% CI)	0.66 (0.44–0.97)	3.93 (2.21–6.99)	2.52 (1.51–4.21)	0.46 (0.19–1.07)	5.11 (0.7–43.8)
**ATA (−)**	*p*	**0.045**	**0.0026**	**0.025**	0.18	0.073
	OR (95% CI	0.7 (.049–0.99)	2.39 (1.33–4.3)	1.78 (1.07–2.97)	0.64 (0.33–1.24)	4.48 (0.75–26.9)
**ACA (+)**	*p*	0.43	**0.0026**	0.4	0.13	**0.001**
	OR (95% CI)	0.78 (0.39–1.52)	3.51 (1.34–8.83)	1.5 (0.51–4.12)	0.24 (0.01–1.66)	12.95 (1.28–130)
**ACA (−)**	*p*	**0.013**	**2.6×10^−5^**	**0.00026**	**0.036**	0.076
	OR (95% CI	0.68 (0.5–0.92)	2.88 (1.72–4.8)	2.21 (1.43–3.42)	0.52 (0.28–0.97)	4.13 (0.75–22.6)
**PF (+)**	*p*	**0.0095**	**<10^−7^**	**3.3×10^−4^**	**0.028**	0.13
	OR (95% CI)	0.65 (0.46–0.91)	3.86 (2.27–6.6)	2.25 (1.4–3.62)	0.47 (0.22–0.97)	3.64 (0.5–31.14)
**PF (−)**	*p*	0.52	0.79	0.17	0.7	**0.037**
	OR (95% CI	0.87 (0.55–1.35)	1.14 (0.43–3.02)	1.62 (0.82–3.2)	0.86 (0.38–1.92)	6.22 (0.87–44.5)

* PF =  pulmonary fibrosis; nominal significance *p* value <0.05; Bonferroni correction for significance was calculated as *p*<0.0013.

Further analysis using dominant model on SSc subsets under only disease condition showed that DPB1*04 carriers including DPB1*04:01 and *04:02 carriers were increased in ACA positive SSc (34.2%) compared to ACA negative SSc (15.4%). DPB1*03:01 carriers were increased in SSc with PF (19.6%) compared to SSc without PF (6.4%) (Table 3). Moreover, examining recessive model on SSc subsets showed that homozygous DPB1*05:01 was decreased in ACA positive SSc (7.9%) compared to ACA negative SSc (23.1%) (Table 3). All of these changes passed a nominal significance level.

**Table 3:** Differentiation of SSc subsets and clinical outcomes with specific HLA-DPB1 alleles.

Alleles	N (%)	N (%)	*p*	OR (95% CI)
**Heterozygous**				
	**ACA (+)**	**ACA (−)**		
DPB1*04	13 (34.2)	36 (15.4)	0.0051	2.86 (1.25–6.49)
DPB1*04:01	9 (23.7)	25 (10.7)	0.025	2.59 (1.01–6.55)
DPB1*04:02	4 (10.5)	11 (4.7)	0.15	2.39 (0.6–8.76)
	**PF (+)**	**PF (−)**		
DPB1*03:01	39 (19.6)	5 (6.4)	0.0069	3.56 (1.27–10.73)
**Homozygous**				
	**ACA (+)**	**ACA (−)**		
DPB1*05:01	3 (7.9)	54 (23.1)	0.033	0.29 (0.07–1.02)

Notes: DPB1*04 only occurred in heterozygotes, heterozygous DPB1*05:01 did not show any association with SSc subsets.

Since HLA-DQB1*05:01 and*06:11 alleles were associated with Chinese SSc in previous studies [14], we examined potential linkage disequilibrium (LD) between these two alleles and the SSc-associated HLA-DPB1 alleles. Both HLA-DQB1*05:01 and*06:11 were not found in LD with either one of HLA-DPB1*03:01, *13:01, *02:01, *17:01 and *35:01. On the other hand, a previously reported SSc-associated HLA-DQB1*03:01 in US population [7] was found to coexist with HLA-DPB1*13:01 in associated with SSc in this Chinese population (12.06% in SSc patients vs. 2.2% in controls, p = 0.0045, OR = 3.98, 95% CI = 1.34–12.54).

## Discussion

SSc is a complex genetic disease. Although multiple genetic loci and genes have been reported in association with SSc, recent GWAS indicated HLA genes conferring the major susceptibility to SSc [6], [15]. The Korean GWAS of SSc reported that HLA-DPB1 contained the strongest risk loci and alleles (DPB*13:01 and *09:01) to SSc [6]. In fact, observed SSc-risk HLA-DPB1 alleles were partially consistent with the studies of SSc in US and UK Caucasians, South Africans and Japanese [7]–[10]. However, different ethnic populations implicated some distinct associations of HLA-DPB1 alleles with SSc. Studies of a Chinese cohort herein demonstrated that HLA-DPB1*13:01 was significantly increased in SSc of Han Chinese, which was consistent with the reports of SSc studies in Korean, South Africans, US and UK Caucasians. In addition, HLA-DPB1*03:01 also appeared as a strong risk allele to Han Chinese SSc, along with an increased frequency of HLA-DPB1*35:01 and decreased *02:01 and *17:01 that achieved a nominal significance of association with SSc. However, the latter observations were not reported in SSc patients of other ethnic populations. On the other hand, HLA-DPB1*09:01 did not show change in frequency in SSc of the Chinese cohort, which is consistent with SSc studies of South Africans [9], US and UK Caucasians [7], [8]. It is worth noting that studies of Africa Americans and Hispanics of US cohorts failed to show any HLA-DPB1 associations with SSc, in which limited study subjects might have impact [7].

Importantly, HLA-DPB1*13:01 and *03:01 also were significantly associated with several subsets of SSc compared to the controls. In particular, increased frequencies of both alleles were strongly associated with dcSSc, ATA positive, ACA negative SSc and SSc with PF. Of notes, associations of DPB1*13:01 with dcSSc and ATA positive SSc also were reported in SSc patients of South Africans and of US/UK Caucasians, respectively [7], [8]. These observations may suggest potential role of these two alleles in severe cases of SSc, and which is in contrast with the previously reported DQB1*05:01 that was strongly associated with ACA positive SSc of Han Chinese [14].

However, comparisons between SSc subsets and controls may not clearly distinguish the association of the alleles with specific subsets of SSc from the association of the alleles with SSc disease in general. A comparison between subsets with and without a specific phenotype may be better to reveal genetic contribution to specific subsets of SSc. Although all of such comparisons did not achieve significance on Bonferroni correction, which is highly conservative in considering a relatively small sample groups of SSc subsets. Nominal significance (p<0.05) may be important in differentiating SSc with and without specific clinical and serological outcomes. Under this criteria, DPB1*04, especially DPB1*04:01 appeared as a risk marker for ACA positive SSc. SSc patients carrying DPB1*03:01 may be more likely to develop PF. Moreover, SSc patients with homozygous DPB1*05:01 may be protect from ACA production (Table 3). These observations were not reported previously. Although the study subjects were relatively large compared to the reports from other ethnic populations [7]–[10], verification studies in other cohorts may be necessary.

In summary, this is the first report of studies of HLA-DPB1 in Chinese SSc. It revealed complex genetic aspects of SSc. Associations of some specific HLA-DPB1 alleles with Chinese SSc were not reported in studies of other ethnic populations. In fact, previous studies of different populations already implicated ethnic differences in genetic association with SSc. Our studies further indicated that even within Asian populations, Chinese SSc appeared some distinct HLA-DPB1 associations from Korean and Japanese. Therefore, genetic heterogeneity among ethnicities may significantly impact the complex trait of SSc. On the other hand, significantly increased DPB1*13:01 in Chinese SSc patients also is a common risk allele to SSc in several ethnic populations including Caucasian, South African, Korean and Han Chinese. Therefore, different ethnic populations also share some genetic determinants of SSc.
